# Comparison of antibiotic and acyclovir usage before and after the implementation of an on-site FilmArray meningitis/encephalitis panel in an academic tertiary pediatric hospital: a retrospective observational study

**DOI:** 10.1186/s12887-020-1944-2

**Published:** 2020-02-05

**Authors:** Alexandra Hagen, Anna Eichinger, Melanie Meyer-Buehn, Tilmann Schober, Johannes Huebner

**Affiliations:** Division of Pediatric Infectious Disease, Hauner Children’s Hospital, University of Munich (LMU), Lindwurmstraße 4, 80337 Munich, Germany

**Keywords:** FilmArray, mPCR, Meningitis, Encephalitis, Meningoencephalitis, Antimicrobial stewardship

## Abstract

**Background:**

Prompt initiation of empiric therapy is common practice in case of suspected meningitis or encephalitis. However, in children the most common pathogens are viruses that usually do not require and are not covered by the applied anti-infective treatment. Novel multiplex PCR (mPCR) panels provide rapid on-site diagnostic testing for a variety of pathogens. This study compared empiric antibiotic and acyclovir usage before and after the introduction of an on-site FilmArray Meningitis/Encephalitis Panel (FA ME Panel).

**Methods:**

We retrospectively compared data for empiric antibiotic and acyclovir usage between pediatric patients with suspected central nervous system (CNS) infection receiving mPCR testing and a matched historical control group. Patients were matched by age and suspected CNS infection. We included all patients for whom empiric antibiotics and/or acyclovir were prescribed.

**Results:**

Each study group consisted of 46 patients with 29 (63.0%) infants and 17 (37.0%) older children. A viral pathogen was diagnosed in 5/46 (10.9%) patients in the control group (all enteroviruses) and in 14/46 (30.4%) patients in the mPCR group (enterovirus *n* = 9; human herpesvirus 6 (HHV-6) *n* = 5), (*p* = 0.038)). Length of Therapy (LoT) and Days of Therapy (DoT) for antibiotics were significantly lower for infants (4.0 vs. 3.0, p = 0.038 and 8.0 vs. 6.0, *p* = 0.015, respectively). Acyclovir therapy was significantly shorter for both, infants and older children (3.0 vs. 1.0 day, *p* < 0.001 for both age groups).

**Conclusion:**

The findings of our study suggest that the introduction of a FA ME Panel into clinical routine procedures is associated with a significantly reduced LoT and DoT of empiric anti-infective treatment in children with suspected meningoencephalitis. The largest effect was observed in infants.

## Background

Acute meningoencephalitis can be caused by a variety of pathogens. In case of bacterial or herpes simplex virus (HSV) infection, early initiation of antibiotics or acyclovir is essential and associated with better outcomes [1–4]. However, in febrile infants undergoing evaluation for meningoencephalitis, the most common infectious agents found are viruses other than HSV, which usually cause self-limiting diseases, do not require anti-infective therapy and are not affected by the treatment with antibiotics and/or acyclovir [5].

It is often difficult to clearly differentiate between the disease-causing organisms using clinical or laboratory information (such as cerebrospinal fluid (CSF) cell counts, inflammatory markers etc.). Particularly in young infants, symptoms are often unspecific and may overlap, [5, 6] while no reliable biomarkers for diagnosing bacterial infections are available [7]. Therefore, early initiation of anti-infective therapy is common practice, [8, 9] resulting in unnecessary usage of antimicrobials. However, parenteral antibiotic and acyclovir administration can lead to serious adverse effects, such as catheter-associated complications, [10] and side effects, such as allergic reactions, [11] diarrhea [12] and nephrotoxicity [13]. In addition, antibiotic-associated changes in the child’s microbiome have been shown to have sometimes long term consequences on the patient’s health [14].

New molecular methods, such as multiplex PCR (mPCR) tests, have been increasingly introduced into clinical routine procedures to allow for simultaneous and more rapid testing for a variety of pathogens. Several authors have already suggested a positive effect of a FilmArray Meningitis/Encephalitis Panel (FA ME Panel) (i.e. mPCR) on empiric treatment. Quick verification or exclusion of the presence of organisms may enable clinicians to early optimize antimicrobial therapy and hence possibly reduce therapy-associated complications and healthcare costs [15–18]. In a previous study conducted at our institution we retrospectively analyzed all patients receiving mPCR testing over the period of one year [19]. To further investigate the impact of the FA ME Panel on empiric antibiotic and acyclovir usage in children with suspected meningoencephalitis, we decided to perform a retrospective observational study using a historical control group of patients prior to the implementation of the FA ME Panel.

## Methods

### Study population

This study is a single center, retrospective observational study conducted at the Dr. von Hauner Children’s Hospital, an academic tertiary care center at the Ludwig-Maximilians-University (LMU) in Munich, Germany. We compared empiric antibiotic and acyclovir usage between patients receiving CSF mPCR panel testing (06/2016 to 02/2017) and a matched historical control group of the last four years (01/2012 to 05/2016) before the introduction of this new method in June 2016.

We identified all patients below 18 years with suspected central nervous system (CNS) infection who underwent mPCR testing during the study period, and for whom empiric antibiotics and/or acyclovir was prescribed. Patients were only included, if complete electronic medical records including information on Length of Therapy (LoT) and Days of Therapy (DoT) were available.

Exclusion criteria were as follows: (1) early onset sepsis within the first week of life (days 0–6), (2) patients with suspected ventricular shunt infection, (3) immune-compromising diseases (malignancy, immunodeficiency), (4) patients who were transferred from another hospital with CSF analysis already done and (5) patients for whom treatment had been adjusted because another cause for the presenting symptoms had been identified in the initial work-up (such as urinary tract infection). During the mPCR period, there were only two cases of bacterial meningitis. In both cases, the mPCR was positive the same day as lumbar puncture was done. We excluded all children with proven bacterial meningitis from further analysis. Cases with traumatic lumbar punctures (i.e. > 500 red blood cells per mm^3^) [20] were excluded from the analysis of CSF values, but were included in the rest of the analyses.

The study group was divided into two groups according to age (i.e. infants < 1 year and older children ≥1 year) and patients were classified according to the suspected CNS infection (i.e. meningitis, meningoencephalitis, encephalitis) by the clinical diagnosis from the discharge summary; all cases were independently reviewed by an infectious disease specialist (JH).

For the control group the above-mentioned inclusion and exclusion criteria and classification were maintained. For each mPCR patient that was included in our study, a historical control patient was selected. To find suitable matches we created a list that included all patients that had received a CSF analysis in the bacteriological laboratory from 2012 to 2016 before the introduction of the mPCR. Patients undergoing lumbar puncture for suspicion of CNS infection were matched by age and suspected CNS infection. For infants, historical controls of +/− two months of the case’s age and for children ≥1 year, historical controls of +/− six months were accepted. To avoid any patient selection bias, we chose the control patient whose age was closest to the case’s age and fulfilled the same suspected CNS infection. A control was only used once for matching. To avoid bias, the process of patient selection and matching has been determined prior to the start of the study.

### Acquired data

Electronic medical records were reviewed to obtain demographic and clinical data. These included sex, height, weight, age, length of stay (LOS), intensive care unit (ICU) stay, diagnosis, symptoms, intravenous antimicrobial therapy during hospital stay and laboratory results (blood, CSF). Values for inflammation markers, including C-reactive protein (CRP), leucocyte count and Interleukin-6 (IL-6) within 24 h up to spinal tap were analyzed. Standard microbiological methods were used for blood and CSF cultures for both study groups (i.e. mPCR and control group). LOS was measured as time from hospitalization to discharge in calendar days. If a patient was hospitalized more than once due to suspicion of meningitis/meningoencephalitis/encephalitis, every stay that occurred independently of the others was included in the analysis. Enteroviral season was defined as June 1st until 31st of October [20].

### Analysis of antibiotic and acyclovir usage

Antibiotic usage was analyzed in Length of Therapy (LoT) and Days of Therapy (DoT). LoT describes the overall period in which a patient receives antibiotic treatment, irrespective of the amount of antibiotics administered during this time, while DoT accounts for all antibiotic drugs given over a certain period. If a patient receives two antibiotic drugs for four days, LoT is four and DoT eight [21]. For acyclovir usage, DoT is identical to LoT. The duration of antibiotic and acyclovir therapy was defined as calendar days from the first to the last dose administered. If the application of anti-infective agents were avoided due to rapid diagnostic testing, LoT and DoT were recorded as zero. During the entire study period (i.e. 2012 to 2017) the recommendations regarding treatment of CNS infections were the same. Although there was an antibiotic stewardship committee program introduced in 2012, the empiric treatment of meningitis or encephalitis was not changed.

### Multiplex PCR

The FA ME Panel (BioMerieux) detects 14 pathogens in CSF in about an hour (assay duration): cytomegalovirus, enterovirus, HSV types 1 and 2, human herpesvirus 6 (HHV-6), human parechovirus, varicella zoster virus, Cryptococcus neoformans/gattii, *Escherichia coli* K1, *Haemophilus influenzae*, *Listeria monocytogenes*, *Neisseria meningitidis*, *Streptococcus agalactiae* and *Streptococcus pneumoniae* [22]. The overall agreement of the FA ME Panel when compared to conventional microbiological procedures is described to be 90.9 to 99.8% [15, 23–25]. A prospective study including 1560 CSF specimens by Leber et al. found a sensitivity ranging between 85 and 100%, depending on the pathogen, while the specificity was ≥99.2% [25]. mPCR tests were performed during opening hours of our on-site bacteriological laboratory (Monday to Friday 8 am to 4 pm and on weekends 10 am to 12 pm) according to the manufacturer’s protocol (BioMerieux; FA ME Panel) [22]. Prior to its implementation at the study site, CSF used to be sent out to the central virology laboratory for viral PCR testing, which usually took between two and five days to obtain results. The testing at the central microbiology and virology laboratory was custom-made for viruses, such as HSV-1/2, enterovirus and HHV-6. Patients with suspected CNS infection were routinely tested for HSV-1/2. However, no specific guidelines and restrictions were in place for clinicians regarding microbiological testing and therefore practice was at the clinician’s discretion. There was no singleplex on-site testing available.

### Statistical analysis

We used the exact Mann-Whitney-U-Test to compare distributions of quantitative variables between independent groups and Fisher’s exact test for categorical variables. Quantitative data are described by median (M) and interquartile range (IQR). Categorical data are presented as absolute number/ total (percentage) [n/N (%)]. All statistical tests and figures were conducted using SPSS Statistics, version 24 (SPSS Inc., Chicago, Ill., USA) and Microsoft Excel. All statistical tests were performed two-sided and a significance level of 5% was used.

## Results

### Demographic and clinical data

**Fig. 1:**
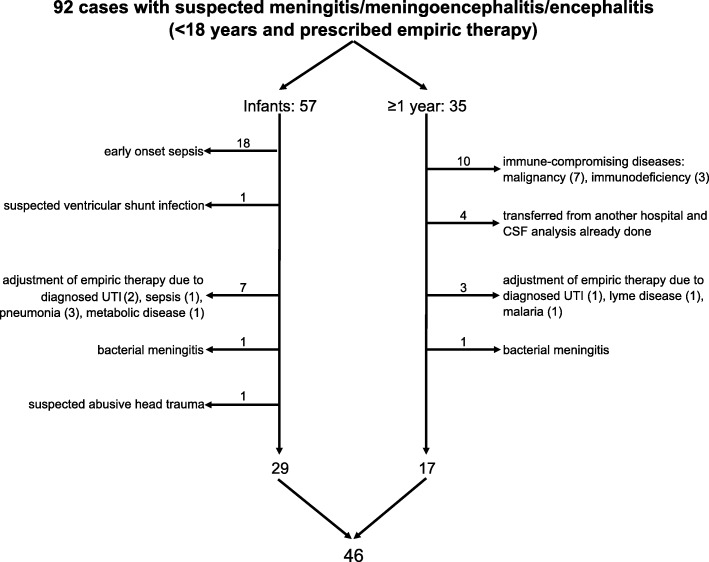
Description of the mPCR study group. Abbreviations: multiplex PCR (mPCR), urinary tract infection (UTI), cerebrospinal fluid (CSF)

During the mPCR period 92 cases fulfilled inclusion criteria. Of these 45/92 (48.9%) cases were not included due to the described exclusion criteria. In addition, one patient with subdural hematoma and suspected abusive head trauma was excluded. This resulted in 46 cases that met study criteria and were enrolled in the study (Fig. 1). One patient in the mPCR group was admitted to the hospital twice due to suspected meningoencephalitis. The admissions took place on two separate occasions with a period of 19 weeks between the two episodes and this child is represented with two different cases.

**Fig. 2:**
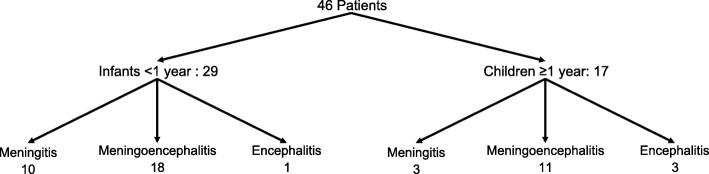
Classification of patients according to age and suspected CNS infection. Abbreviations: central nervous system (CNS)

The classification of patients according to age and suspected CNS infection is shown in Fig. 2. Each study group consisted of 29/46 (63.0%) infants and 17/46 (37.0%) older children. Most infants were less than three months old, with 23/29 (79.3%) in the control and 24/29 (82.8%) in the mPCR group (*p* = 1.000). Per study group a total of 13/46 (28.3%) patients with suspected meningitis, 29/46 (63.0%) with suspected meningoencephalitis and 4/46 (8.7%) with suspected encephalitis were included in the analysis.

Demographics, LOS, ICU stay, number of proven viral CNS infections and laboratory results for each study group are summarized in Table 1. No significant differences (*p* > 0.05) were found between the study groups, except for the number of proven viral CNS infections (see below). There were no significant differences in symptoms between the two study groups (see Additional file 1: Table S1). The most common symptom in both study groups was fever (42/46 (91.3%) in the control vs. 41/46 (89.1%) in the mPCR group).

**Table 1:** Comparison of demographic and clinical data between the control and mPCR group

	Control group (***n*** = 46)	mPCR group (n = 46)	***p*** value
Age (m)	**3.1** (1.1–22.1)	**2.8** (1.2–24.5)	
Sex: male	**27/46** (58.7%)	**26/46** (56.5%)	1.000
Weight (kg)	**6.1 (**4.3–11.6)	**6.0** (4.4–11.9)	0.903
LOS (d)	**5.0** (5.0–6.0)	**5.0** (4.0–7.0)	0.384
ICU	**10/46** (21.7%)	**11/46** (23.9%)	1.000
CNS infections	**5/46** (10.9%)	**14/46** (30.4%)	0.038
CRP (mg/dl)	**0.6** (0.1–1.7)	**0.8** (0.3–2.2)^1^	0.267
Leucocytes (G/L)	**8.4** (6.3–14.0)	**9.0** (7.2–14.1)^2^	0.577
IL-6 (pg/ml)	**30.0** (14.9–63.7)^3^	**50.1** (10.2–106.5)^4^	0.401
CSF samples analyzed	**36/46** (78.3%)	**33/46** (71.7%)	
Cell count (/μl)	**2.0** (1.0–5.5)	**2.0** (1.0–6.0)	0.522
Protein (mg/dl)	**34.4** (19.4–53.7)	**33.3** (17.6–56.2)	0.936
Glucose (mg/dl)	**56.6** (52.0–69.6)	**56.7** (50.5–66.0)	0.893

The exact Mann-Whitney-U-Test was used to compare distributions of quantitative variables between independent groups and the Fisher’s exact test for categorical variables. Quantitative data are described by median (**M**) and interquartile range (IQR). Categorical data are presented as absolute number/ total (percentage) [**n/N** (%)]

Abbreviations: multiplex PCR (mPCR), months (m), days (d), length of stay (LOS), intensive care unit (ICU), central nervous system (CNS), C-reactive protein (CRP), Interleukin-6 (IL-6), cerebrospinal fluid (CSF)

Data available for ^1^ 45 patients, ^2^ 43 patients, ^3^ 34 patients, ^4^ 32 patients

### Detected pathogens

**Fig. 3:**
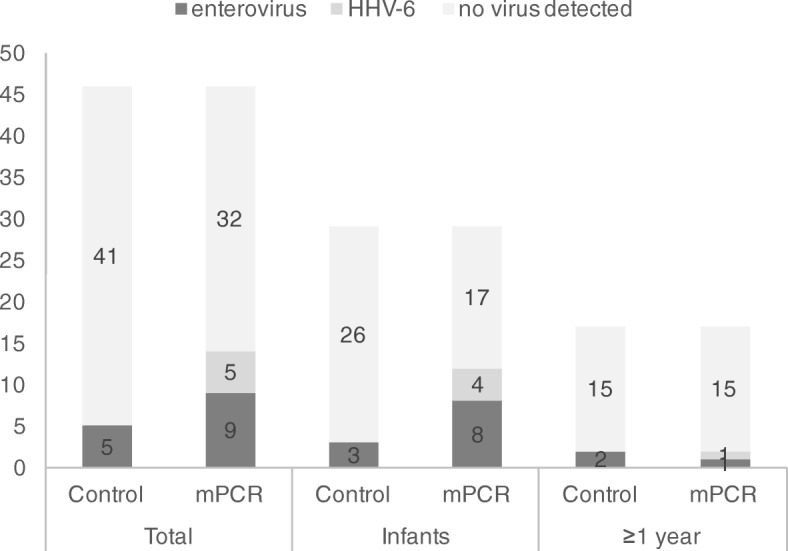
Number of patients with detected enteroviruses and HHV-6 for both study groups and by age group. Abbreviations: human herpesvirus 6 (HHV-6), multiplex PCR (mPCR)

Overall, a viral pathogen was detected in CSF in 5/46 (10.9%) patients in the control group and in 14/46 (30.4%) patients in the mPCR group (*p* = 0.038, Table 1, Fig. 3). In the control group only enteroviruses were found and in the mPCR group these accounted for 9 (64.3%) of the 14 cases, while in the remaining 5 (35.7%) patients HHV-6 was identified. In both study groups, most detected pathogens were found in infants (3/5 (60.0%) in the control vs. 12/14 (85.7%) in the mPCR group) (see Fig. 3) of which all, except for one per study group, were isolated in children younger than three months.

Looking at the number of tests ordered for these viral pathogens in the control group, it amounts to 18/46 (39.1%) tests for enteroviruses (8 in infants and 10 in older children) and 2/46 (4.3%) tests for HHV-6, both performed in children above the age of one year (Table 2). Hence, while the absolute number of patients with detected enteroviruses is higher in the mPCR group than in the control group (9/46 (19.6%) in the mPCR vs. 5/46 (10.9%) in the control group, *p* = 0.385), the relative number in relation to the number of patients receiving enteroviral PCR testing is lower (9/46 (19.6%) in the mPCR vs. 5/18 (27.8%) in the control group, *p* = 0.512). The children in the control group were not all tested for the viral pathogens included in the FA ME Panel. Table 2 summarizes the testing for viral pathogens in CSF in both study groups.

**Table 2:** PCR testing for viral pathogens in CSF in both study groups

	Control group	mPCR group	p value
Viral pathogens included in the FA ME Panel			
Cytomegalovirus	**3/46** (6.5%)	**46/46** (100.0%)	< 0.001
Infants	**1/29** (3.4%)	**29/29** (100.0%)	< 0.001
≥ 1 year	**2/17** (11.8%)	**17/17** (100.0%)	< 0.001
Enterovirus	**18/46** (39.1%)	**46/46** (100.0%)	< 0.001
Infants	**8/29** (27.6%)	**29/29** (100.0%)	< 0.001
≥ 1 year	**10/17** (58.8%)	**17/17** (100.0%)	0.007
Herpes simplex virus 1/2	**43/46** (93.5%)	**46/46** (100.0%)	0.242
Infants	**26/29** (89.7%)	**29/29** (100.0%)	0.237
≥ 1 year	**17/17** (100.0%)	**17/17** (100.0%)	–
Human herpesvirus 6	**2/46** (4.3%)	**46/46** (100.0%)	< 0.001
Infants	**0/29** (0.0%)	**29/29** (100.0%)	< 0.001
≥ 1 year	**2/17** (11.8%)	**17/17** (100.0%)	< 0.001
Human parechovirus	**0/46** (0.0%)	**46/46** (100.0%)	< 0.001
Infants	**0/29** (0.0%)	**29/29** (100.0%)	< 0.001
≥ 1 year	**0/17** (0.0%)	**17/17** (100.0%)	< 0.001
Varicella zoster virus	**3/46** (6.5%)	**46/46** (100.0%)	< 0.001
Infants	**0/29** (0.0%)	**29/29** (100.0%)	< 0.001
≥ 1 year	**3/17** (17.6%)	**17/17** (100.0%)	< 0.001
Other viral pathogens			
Human herpesvirus 7	**3/46** (6.5%)	**0/46** (0 .0%)	0.242
Infants	**0/29** (0.0%)	**0/29** (0.0%)	–
≥ 1 year	**3/17** (17.6%)	**0/17** (0.0%)	0.227
Epstein-Barr virus	**3/46** (6.5%)	**0/46** (0.0%)	0.242
Infants	**3/29** (10.3%)	**0/29** (0.0%)	0.237
≥ 1 year	**0/17** (0.0%)	**0/17** (0.0%)	–
Adenovirus	**3/46** (6.5%)	**1/46** (2.2%)	0.617
Infants	**1/29** (3.4%)	**1/29** (3.4%)	1.000
≥ 1 year	**2/17** (11.8%)	**0/17** (0.0%)	0.485
Influenza A virus	**1/46** (2.2%)	**0/46** (0.0%)	1.000
Infants	**0/29** (0.0%)	**0/29** (0.0%)	–
≥ 1 year	**1/17** (5.9%)	**0/17** (0.0%)	1.000
Influenza B virus	**1/46** (2.2%)	**0/46** (0.0%)	1.000
Infants	**0/29** (0.0%)	**0/29** (0.0%)	–
≥ 1 year	**1/17** (5.9%)	**0/17** (0.0%)	1.000
Measles virus	**1/46** (2.2%)	**0/46** (0.0%)	1.000
Infants	**0/29** (0.0%)	**0/29** (0.0%)	–
≥ 1 year	**1/17** (5.9%)	**0/17** (0.0%)	1.000
Mumps virus	**1/46** (2.2%)	**0/46** (0.0%)	1.000
Infants	**0/29** (0.0%)	**0/29** (0.0%)	–
≥ 1 year	**1/17** (5.9%)	**0/17** (0.0%)	1.000

The Fisher’s exact test was used for categorical variables. Categorical data are presented as absolute number/ total (percentage) [**n/N** (%)]

Abbreviations: cerebrospinal fluid (CSF), multiplex PCR (mPCR), FilmArray Meningitis/Encephalitis Panel (FA ME Panel)

The number of patients being admitted during enteroviral season is similar in both study groups with 21/46 (45.7%) patients (16 infants and 5 older children) in the control vs. 26/46 (56.5%) (19 infants and 7 older children) in the mPCR group (*p* = 0.404).

### Antibiotic and acyclovir usage

In both study groups, antibiotic drugs were prescribed for a total of 42/46 (91.3%) patients and acyclovir for a total of 33/46 (71.7%) patients (Table 3). However, in the mPCR group in three of these children antibiotic and/or acyclovir therapy was not started because results were available on the day of lumbar puncture: two infants younger than three months with suspected meningoencephalitis and one older patient with suspected encephalitis. For both infants, a viral pathogen was found in the CSF before antibiotic therapy was initiated (1 enterovirus and 1 HHV-6). For one of these infants, no acyclovir therapy was administered after receiving the mPCR results, while the other child had already received one dose of acyclovir. The patient older than one year with suspected encephalitis did not receive any acyclovir.

**Table 3:** Comparison of antibiotic and acyclovir usage between the control and mPCR group

	Control group	mPCR group	p value^a^
I. Antibiotic therapy			
Patients with suspicion of Meningitis/ Meningoencephalitis	**42/46** (91.3%)	**42/46** (91.3%)	
Infants	**28/29** (96.6%)	**28/29** (96.6%)	
≥ 1 year	**14/17** (82.4%)	**14/17** (82.4%)	
LoT (antibiotics)	**4.0** (3.0–5.0)	**3.0** (1.0–5.0)	0.028
Infants	**4.0** (3.5–5.5)	**3.0** (2.0–5.0)	0.038
≥ 1 year	**4.0** (3.0–5.0)	**2.5** (1.0–7.0)	0.280
DoT (antibiotics)	**6.0** (4.0–10.0)	**4.0** (2.0–8.0)	0.023
Infants	**8.0** (6.0–11.0)	**6.0** (3.5–10.0)	0.015
≥ 1 year	**4.0** (3.0–5.0)	**2.5** (1.0–7.0)	0.280
II. Acyclovir therapy			
Patients with suspicion of Meningoencephalitis/ Encephalitis	**33/46** (71.7%)	**33/46** (71.7%)	
Infants	**19/29** (65.5%)	**19/29** (65.5%)	
≥ 1 year	**14/17** (82.4%)	**14/17** (82.4%)	
LoT (acyclovir)	**3.0** (3.0–4.0)	**1.0** (1.0–2.0)	< 0.001
Infants	**3.0** (3.0–4.0)	**1.0** (1.0–2.0)	< 0.001
≥ 1 year	**3.0** (3.0–4.0)	**1.0** (1.0–2.0)	< 0.001

The exact Mann-Whitney-U-Test was used to compare distributions of quantitative variables between independent groups. Quantitative data are described by median (**M**) and interquartile range (IQR). Categorical data are presented as absolute number/ total (percentage) [**n/N** (%)]

Abbreviations: multiplex PCR (mPCR), Length of Therapy (LoT), Days of Therapy (DoT)

^a^The p value always refers to the comparison of the number of patients being prescribed either antibiotics or acyclovir in the control and mPCR group

In each study group, there were 28/29 (96.6%) infants and 14/17 (82.4%) older children with suspected meningitis or meningoencephalitis. For most of these infants a combination of ampicillin and third-generation cephalosporin was prescribed: 25/28 (89.3%) in the control and 22/28 (78.6%) in the mPCR group, while for the two infants mentioned above the administration of these antibiotic drugs was avoided due to rapid detection of a viral pathogen. Only one antibiotic drug was administered in 3/28 (10.7%) infants in the control group and in 6/28 (21.4%) infants in the mPCR group. All patients ≥1 year received only one antibiotic drug. Antibiotic therapy was initiated prior to lumbar puncture in three infants in the control group and in two infants in the mPCR group. These prior administrations took place within 24 h before spinal tap. A total of three children, two of the control group and one of the mPCR group, received one dose of intravenous antibiotics before admission through the emergency physician. These pre-hospital administrations were not included in our analysis.

Overall, antibiotic usage (LoT and DoT) was significantly lower (LoT: 4.0 (IQR 3.0–5.0) days in the control vs. 3.0 (IQR 1.0–5.0) days in the mPCR group, *p* = 0.028 and DoT: 6.0 (IQR 4.0–10.0) days in the control vs. 4.0 (IQR 2.0–8.0) days in the mPCR group, *p* = 0.023; Table 3). When stratifying for age, a significant reduction for LoT and DoT of antibiotics was observed for infants (LoT: 4.0 (IQR 3.5–5.5) days in the control vs. 3.0 (IQR 2.0–5.0) days in the mPCR group, *p* = 0.038 and DoT: 8.0 (IQR 6.0–11.0) days in the control vs. 6.0 (IQR 3.5–10.0) days in the mPCR group, *p* = 0.015), while for children ≥1 year no significant differences in antibiotic usage were seen (LoT and DoT: *p* = 0.280).

Herpes simplex was not identified in any patient during the study. Usually, in our hospital we identify herpes simplex in CSF only about 1 time per year. Acyclovir was administered prior to lumbar puncture in one patient in the mPCR group, given within 24 h before spinal tap. LoT of “empiric” acyclovir was significantly shorter for all age groups (3.0 (IQR 3.0–4.0) days in the control vs. 1.0 (IQR 1.0–2.0) day in the mPCR group, *p* < 0.001; Table 3).

## Discussion

In this study, we compared empiric anti-infective usage before and after the implementation of a FA ME Panel in a pediatric hospital. Our data indicate that the introduction of an on-site mPCR into clinical routine procedures is associated with reduced empiric therapy in children with suspected meningoencephalitis. Overall, LoT and DoT of antibiotics and acyclovir were significantly lower. When stratifying for age, a significant reduction for LoT and DoT of antibiotics was only observed for infants, while acyclovir treatment was significantly shorter for both, infants and older children.

Our results suggest that the implementation of rapid molecular testing for meningoencephalitis in pediatric hospitals can lead to earlier optimization of empiric therapy, as seen in several recent studies [26–29]. However, in contrast to our study, these reports refer to single PCR assays. By using a mPCR, this effect might be even greater because of the simultaneous testing for a variety of pathogens. Recent studies by Rogers et al. and Subramony et al. have shown the benefits of combining several etiological organisms in one mPCR for acute respiratory tract diseases [30, 31]. Another study recently published, evaluated the impact of the FA ME Panel on antibiotic therapy in children with confirmed CNS infection by comparing antibiotic usage before and after its introduction. However, only patients with a discharge diagnosis of meningitis or encephalitis were included [32]. In contrast to that study, our study includes all patients with suspected CNS infection and also analyzes the usage of antiviral agents.

During the mPCR period, the majority of pathogens detected in CSF were enteroviruses and HHV-6, that usually cause self-limiting diseases and only need supportive care [33]. This is in line with the published literature where enteroviruses and HHV6 are the most common detected pathogens [25, 34]. There were two cases of bacterial meningitis and no case of HSV encephalitis, confirming that the incidence of these infectious organisms in children is low [35–37]. However, as they are associated with high morbidity and mortality, especially when anti-infective therapy is delayed, prompt initiation of empiric treatment is common practice [1–4]. This often leads to unnecessary usage of antibiotics and acyclovir, associated costs and side effects. Moreover, Gaensbauer et al. noticed an increase in acyclovir usage, while no increase in HSV diseases was observed, [37] hence, further highlighting the necessity for rapid diagnostic testing. It has been shown that PCR results may be negative very early in HSV encephalitis [38]. Thus, in patients with high suspicion of HSV infection due to clinical findings and anamnesis, careful interpretation of a negative HSV PCR result is required before discontinuing empiric acyclovir therapy. In these cases a second lumbar puncture and repeated testing might be necessary.

The biggest difference between both study groups regarding anti-infective usage was observed in infants. In this age group most viral pathogens were found and LoT and DoT of antibiotics and acyclovir, were significantly lower. In each study group, most infants were younger than three months old. These patients often present with unspecific symptoms and usually receive empiric therapy while undergoing several diagnostic procedures, including lumbar puncture [5]. In older patients, symptoms are more specific and thus these can be managed without anti-infective treatment more often [39]. Moreover, the highest incidence of bacterial meningitis was found to be in infants below the age of six months [40]. Therefore, additional rapid molecular testing may be of greater benefit for young infants.

The implementation of our in-house mPCR has facilitated more rapid pathogen detection, while covering a broad range of infectious organisms and raising awareness for other viral agents. Prior to the implementation of the FA ME Panel, tests for viruses other than HSV were rarely ordered. This lack of awareness for other viral agents was also seen in other institutions [16, 41–43]. Hence, many viral CNS infections are likely to have remained undetected, resulting in unnecessary continuation of anti-infective therapy. Since the introduction of the FA ME Panel, all patients with suspected meningoencephalitis are tested for 14 different pathogens, including 7 viruses, 6 bacteria and a yeast. Moreover, in our institution testing for viral pathogens used to be sent out to a reference laboratory, usually taking between two and five days to receive results. By implementing an on-site PCR, results are available sooner than before, hence enabling clinicians to earlier adapt therapy procedures. Thus, it is likely that the increase in the number of detected viral pathogens and the reduction in anti-infective therapy seen in our study are a result of both, frequent testing for a wider variety of pathogens and more rapid pathogen detection. However, during the mPCR period only two of seven possible viral pathogens included in the mPCR were detected. This might raise the question if using in-house PCR assays for HSV, enterovirus and HHV-6 instead of using the mPCR, may be more cost effective in some institutions. Some authors even implied that the sensitivity of a singleplex assay to detect viral agents is greater than that of a mPCR [15, 17, 44]. However, this approach involves the risk of missing a viral pathogen and requires a certain level of medical experience. To further investigate this questioning, additional prospective (multicenter) studies with longer time periods are needed to possibly include more detected infectious agents.

In our study, we found a significant difference in the number of patients with confirmed viral CNS infection (5/46 (10.9%) in the control vs. 14/46 (30.4%) in the mPCR group, *p* = 0.038). The number of patients being admitted during enteroviral season was similar in both study groups (21/46 (45.7%) in the control and 26/46 (56.5%) in the mPCR group, *p* = 0.404). Thus, comparisons regarding enterovirus results and testing orders are not biased by seasonality. Comparing the relative frequency of patients with detected enteroviruses in relation to the number of patients receiving enteroviral PCR testing between both study groups, a higher percentage can be seen in the control group (27.8% in the control vs. 19.6% in the mPCR group). However, the overall higher detection rate of enteroviruses after the implementation of the mPCR (10.9% in the control vs. 19.6% in the mPCR group) underlines that a targeted approach using singleplex assays involves the risk of missing viral agents. In previous studies, rapid detection of enteroviruses in pediatric patients was already shown to be associated with reduced antibiotic usage [20, 29, 39, 45]. Hence, the higher detection rate of enteroviruses after the implementation of the mPCR, is likely to have contributed to the reduction in anti-infective therapy seen in our study.

In contrast to the mPCR group, no HHV-6 was found in the control group. We suggest this being mainly due to the limited number of tests being ordered for HHV-6 by clinicians (*n* = 2). However, the role of this viral agent regarding CNS infections remains unclear. HHV-6-positivity may represent a primary infection, a latent state of infection, a reactivation or chromosomal integration [25, 46]. Hence terminating empiric anti-infective therapy based on a positive HHV-6 result in CSF alone is not appropriate. The significance of HHV-6 positivity should be interpreted in the context of the patient, including clinical symptoms, immune status, laboratory results and cranial imaging [25, 46].

Some authors have suggested that the positive impact of rapid testing on antibiotic and acyclovir reduction may be even increased with faster turn-around-time [26, 29, 39, 47]. In our study rapid verification of a viral pathogen by the mPCR enabled clinicians to withhold anti-infective therapy for two infants, as results had been available prior to administration (1 enterovirus and 1 HHV-6). In another case acyclovir therapy had not been initiated due to a mPCR result being negative for HSV shortly after admission. These findings demonstrate that by faster turn-around-time, antibiotic and acyclovir administration can be completely avoided, as previously seen in a study by Van et al. [26] Our mPCR is not run outside the microbiology laboratory working hours. If it was run 24 h a day, 7 days a week, its potential influence on empiric therapy might be even greater [26]. However, it is only feasible to withhold anti-infective treatment if the patient is clinically stable and the CSF cell count is either normal or moderately increased [48]. Due to the often rapid course of meningoencephalitis we hospitalize these children and monitor them until CSF culture results are negative after 48 h of incubation. Furthermore, despite faster PCR turnaround times, several difficulties might be encountered in the clinical setting that make it difficult for pediatricians to completely withhold anti-infective therapy. Pediatric patients, especially infants, often present with unspecific symptoms that make it difficult to distinguish between viral and bacterial infection and there is always the risk for false positive or negative results [5, 6, 22]. In addition, the detection of a viral infection does not rule out a concomitant bacterial infection [16].

We did not observe a significant reduction in length of stay after the introduction of the FA ME Panel into our clinical routine setting. Despite the significant higher amount of proven viral CNS infections for infants in the mPCR group (12/29 (41.4%) in the mPCR vs. 3/29 (10.3%), *p* = 0.015 in the control group), median hospital LOS for infants was 6.0 days in both study groups. As previously described by Archimbaud et al. [39], enterovirus positive infants were often not immediately discharged after pathogen detection. These infants are kept for observation until having recovered and appearing clinically well.

Our study has several limitations. First, this is a single center study, which means that our results may not be representative of other hospitals. Second, the sample size was small. Third, by its retrospective nature, there is always the risk for information bias and missing data. Moreover, by excluding patients with bacterial meningitis, we cannot make any conclusions on the impact of the FA ME Panel for these children. During the mPCR period, only two bacterial CNS infections were detected. In both cases the mPCR was positive for bacterial pathogens the same day as lumbar puncture and confirmed by conventional culture. In both cases, the positive result has not changed the empiric therapy, and culture confirmation is necessary to assess antimicrobial susceptibility to optimize treatment. Furthermore, we focused on patients with high suspicion of CNS infection, hence all were receiving anti-infective therapy. Patients, for whom bacterial or HSV meningoencephalitis was excluded based on presenting symptoms or CSF analysis were not included in our study. In these cases, the mPCR cannot influence empiric therapy, and is therefore of lesser importance in these situations. For 32/46 (69.6%) children the FA ME Panel showed negative results. Confirmatory testing by singleplex PCR was only performed in 8/32 (25.0%) patients. All of these were tested for HSV and 7 of these also for enterovirus. All were confirmed negative. However, for the other patients with FilmArray negative samples, no confirmatory testing was performed.

## Conclusions

To summarize, our study provides data regarding the potential influence of a FA ME Panel on anti-infective therapy after its implementation in a pediatric setting. Our results suggest that in children with suspected meningoencephalitis that are treated with empiric antibiotics and/or acyclovir, rapid results provided by a mPCR can possibly reduce anti-infective therapy or prevent treatment in the first place.

## Supplementary information

**Additional file 1: Table S1.** Comparison of symptoms between the control and mPCR group. The Fisher’s exact test was used for categorial variables. Categorial data are presented as absolute number/total (percentage) [n/N (%)]. Abbreviations: multiplex PCR (mPCR), seconds (s). * including reduced vigilance, drowsiness, lethargy, apathy, disorientation, personality change. ** including photophobia, auditory hypersensitivity, hypersensitivity to touch, irritability

## Data Availability

The datasets used during the current study are available from the corresponding author on reasonable request.

## Abbreviations

CNS: Central nervous system
CRP: C-reactive protein
CSF: Cerebrospinal fluid
DoT: Days of Therapy
FA ME Panel: FilmArray Meningitis/Encephalitis Panel
HHV-6: Human herpesvirus 6
HSV: Herpes simplex virus
ICU: Intensive care unit
IL-6: Interleukin-6
LOS: Length of stay
LoT: Length of Therapy
mPCR: Multiplex PCR
UTI: Urinary tract infection
