# Supplementary material for: Comparison of antibiotic and acyclovir usage before and after the implementation of an on-site FilmArray meningitis/encephalitis panel in an academic tertiary pediatric hospital: a retrospective observational study
Source: BMC Pediatr. 2020 Feb 5;20:56. doi: 10.1186/s12887-020-1944-2 (PMC7001287; doi:10.1186/s12887-020-1944-2)
Supplement: Supplementary file 1 — Additional file 1: Table S1. Comparison of symptoms between the control and mPCR group. The Fisher’s exact test was used for categorial variables. Categorial data are presented as absolute number/total (percentage) [n/N (%)]. Abbreviations: multiplex PCR (mPCR), seconds (s). * including reduced vigilance, drowsiness, lethargy, apathy, disorientation, personality change. ** including photophobia, auditory hypersensitivity, hypersensitivity to touch, irritability [file 12887_2020_1944_MOESM1_ESM.docx]

Additional table file 1: Comparison of symptoms between the control and mPCR group.

|  | **Control group** | **mPCR group** | **p value** |
| --- | --- | --- | --- |
| Seizure | **14/46** (30.4%) | **14/46** (30.4%) | 1.000 |
| Infants | **3/29** (10.3%) | **3/29** (10.3%) | 1.000 |
| ≥1 year | **11/17** (64.7%) | **11/17** (64.7%) | 1.000 |
| Nuchal rigidity | **7/46** (15.2%) | **5/46** (10.9%) | 0.758 |
| Infants | **0/29** (0.0%) | **1/29** (3.4%) | 1.000 |
| ≥1 year | **7/17** (41.2%) | **4/17** (23.5%) | 0.465 |
| Headache | **5/46** (10.9%) | **4/46** (8.7%) | 1.000 |
| Infants | **0/29** (0.0%) | **0/29** (0.0%) | --- |
| ≥1 year | **5/17** (29.4%) | **4/17** (23.5%) | 1.000 |
| Altered mental status* | **12/46** (26.1%) | **15/46** (32.6%) | 0.647 |
| Infants | **4/29** (13.8%) | **8/29** (27.6%) | 0.331 |
| ≥1 year | **8/17** (47.1%) | **7/17** (41.2%) | 1.000 |
| Hypersensitivity** | **6/46** (13.0%) | **9/46** (19.6%) | 0.574 |
| Infants | **4/29** (13.8%) | **8/29** (27.6%) | 0.331 |
| ≥1 year | **2/17** (11.8%) | **1/17** (5.9%) | 1.000 |
| Bulging fontanel | **2/46** (4.3%) | **3/46** (6.5%) | 1.000 |
| Infants | **2/29** (6.9%) | **3/29** (10.3%) | 1.000 |
| ≥1 year | **0/17** (0.0%) | **0/17** (0.0%) | --- |
| Poor feeding | **12/46** (26.1%) | **19/46** (41.3%) | 0.185 |
| Infants | **11/29** (37.9%) | **18/29** (62.1%) | 0.114 |
| ≥1 year | **1/17** (5.9%) | **1/17** (5.9%) | 1.000 |
| Fever | **42/46** (91.3%) | **41/46** (89.1%) | 1.000 |
| Infants | **26/29** (89.7%) | **24/29** (82.8%) | 0.706 |
| ≥1 year | **16/17** (94.1%) | **17/17** (100%) | 1.000 |
| Hypothermia | **0/46** (0.0%) | **2/46** (4.3%) | 0.495 |
| Infants | **0/29** (0.0%) | **2/29** (6.9%) | 0.491 |
| ≥1 year | **0/17** (0.0%) | **0/17** (0.0%) | --- |
| Capillary Refill Time >3s | **6/46** (13.0%) | **3/46** (6.5%) | 0.485 |
| Infants | **5/29** (17.2%) | **3/29** (10.3%) | 0.706 |
| ≥1 year | **1/17** (5.9%) | **0/17** (0.0%) | 1.000 |
| Pale skin | **15/46** (32.6%) | **18/46** (39.1%) | 0.664 |
| Infants | **12/29** (41.4%) | **15/29** (51.7%) | 0.599 |
| ≥1 year | **3/17** (17.6%) | **3/17** (17.6%) | 1.000 |
| Nausea/ Vomiting | **10/46** (21.7%) | **10/46** (21.7%) | 1.000 |
| Infants | **5/29** (17.2%) | **5/29** (17.2%) | 1.000 |
| ≥1 year | **5/17** (29.4%) | **5/17** (29.4%) | 1.000 |
| Exanthema | **6/46** (13.0%) | **3/46** (6.5%) | 0.485 |
| Infants | **5/29** (17.2%) | **3/29** (10.3%) | 0.706 |
| ≥1 year | **1/17** (5.9%) | **0/17** (0.0%) | 1.000 |
| Petechiae | **1/46** (2.2%) | **0/46** (0.0%) | 1.000 |
| Infants | **0/29** (0.0%) | **0/29** (0.0%) | --- |
| ≥1 year | **1/17** (5.9%) | **0/17** (0.0%) | 1.000 |
| Jaundice | **2/46** (4.3%) | **3/46** (6.5%) | 1.000 |
| Infants | **2/29** (6.9%) | **3/29** (10.3%) | 1.000 |
| ≥1 year | **0/17** (0.0%) | **0/17** (0.0%) | --- |
| Cough/Cold/red throat | **10/46** (21.7%) | **15/46** (32.6%) | 0.349 |
| Infants | **3/29** (10.3%) | **8/29** (27.6%) | 0.179 |
| ≥1 year | **7/17** (41.2%) | **7/17** (41.2%) | 1.000 |

The Fisher’s exact test was used for categorical variables. Categorical data are presented as absolute number/ total (percentage) [**n/N** (%)].

Abbreviations: multiplex PCR (mPCR), seconds (s)

* including reduced vigilance, drowsiness, lethargy, apathy, disorientation, personality change

** including photophobia, auditory hypersensitivity, hypersensitivity to touch, irritability
